# Supplementary material for: Iron derived from autophagy-mediated ferritin degradation induces cardiomyocyte death and heart failure in mice
Source: eLife. 2021 Feb 2;10:e62174. doi: 10.7554/eLife.62174 (PMC7853718; doi:10.7554/eLife.62174)
Supplement: Figure 1—source data 2. — BP, blood pressure; TL, tibia length; LV, left ventricle; LVIDd, end-diastolic left ventricular internal dimension; LVIDs, end-systolic left ventricular internal dimension; IVSd, end-diastolic interventricular septum thickness; LVPWd, end-diastolic left ventricular posterior wall thickness; FS, fractional shortening. The data are the mean ± SEM. n indicates the number of biologically independent samples. The data were evaluated by unpaired, two-tailed Student’s t-test. [file elife-62174-fig1-data2.docx]

**Figure 1—source data 2. Physiological and echocardiographic parameters in 8- to 10-week-old *Ncoa4*^+/+^ and *Ncoa4^–/–^* mice at baseline.**

|  | *Ncoa4^+/+^* (*n* = 8) | | | *Ncoa4^–/–^* (*n* = 8) | | | *P* value |
| --- | --- | --- | --- | --- | --- | --- | --- |
| Body weight (g) | 25.7 | ± | 0.5 | 25.7 | ± | 0.4 | 0.925 |
| Tibia length (mm) | 16.8 | ± | 0.1 | 16.8 | ± | 0.2 | 0.854 |
| Systolic BP (mmHg) | 103 | ± | 2 | 100 | ± | 3 | 0.350 |
| Heart rate (bpm) | 709 | ± | 2 | 712 | ± | 2 | 0.368 |
| Heart weight/TL (mg/mm) | 8.20 | ± | 0.34 | 8.18 | ± | 0.18 | 0.955 |
| LV weight/TL (mg/mm) | 5.71 | ± | 0.27 | 5.79 | ± | 0.13 | 0.792 |
| Lung weight/TL (mg/mm) | 8.94 | ± | 0.23 | 9.06 | ± | 0.21 | 0.700 |
| LVIDd (mm) | 3.13 | ± | 0.04 | 3.08 | ± | 0.04 | 0.360 |
| LVIDs (mm) | 1.55 | ± | 0.03 | 1.52 | ± | 0.02 | 0.384 |
| IVSd (mm) | 0.74 | ± | 0.01 | 0.75 | ± | 0.004 | 0.055 |
| LVPWd (mm) | 0.73 | ± | 0.01 | 0.73 | ± | 0.003 | 0.221 |
| FS (%) | 50.7 | ± | 0.4 | 50.7 | ± | 0.4 | 0.909 |

BP, blood pressure; TL, tibia length; LV, left ventricle; LVIDd, end-diastolic left ventricular internal dimension; LVIDs, end-systolic left ventricular internal dimension; IVSd, end-diastolic interventricular septum thickness; LVPWd, end-diastolic left ventricular posterior wall thickness; FS, fractional shortening. The data are the mean ± SEM. *n* indicates the number of biologically independent samples. The data were evaluated by unpaired, two-tailed Student’s *t*-test. Source Data file is provided in Figure 1 - source data 3.
