# Supplementary material for: Iron derived from autophagy-mediated ferritin degradation induces cardiomyocyte death and heart failure in mice
Source: eLife. 2021 Feb 2;10:e62174. doi: 10.7554/eLife.62174 (PMC7853718; doi:10.7554/eLife.62174)
Supplement: Figure 4—source data 2. — PCV, packed cell volume; MCV, mean corpuscular volume; MCH, mean corpuscular hemoglobin; MCHC, mean corpuscular hemoglobin concentration; CH, hemoglobin concentration. Data are the mean ± SEM. n indicates the number of biologically independent samples. The data were evaluated by one-way analysis of variance (ANOVA), followed by Tukey–Kramer’s post hoc test. *p=0.0418 and **p=0.0108 versus the corresponding sham-operated group. Exact p-values are provided in Supplementary file 1. [file elife-62174-fig4-data2.docx]

**Figure 4—source data 2. Hematological parameters and red cell indices in *Ncoa4*^+/+^ and *Ncoa4^–/–^* mice**.

|  | *Ncoa4*^+/+^ | | | | | | *Ncoa4*^–/–^ | | | | | |
| --- | --- | --- | --- | --- | --- | --- | --- | --- | --- | --- | --- | --- |
|  | Sham | | | TAC | | | Sham | | | TAC | | |
|  | (*n* = 6) | | | (*n* = 6) | | | (*n* = 6) | | | (*n* = 6) | | |
| Hemoglobin (g/dl) | 13.4 | ± | 0.2 | 13 | ± | 0.3 | 13.3 | ± | 0.1 | 12.9 | ± | 0.3 |
| PCV (%) | 43.2 | ± | 1.3 | 41.8 | ± | 1.1 | 42.3 | ± | 0.9 | 41.4 | ± | 1.2 |
| MCV (%) | 47.7 | ± | 1.5 | 48.5 | ± | 0.7 | 47.9 | ± | 1.4 | 48.6 | ± | 1.2 |
| MCH (pg) | 14.8 | ± | 0.1 | 14.9 | ± | 0.1 | 15.1 | ± | 0.1 | 15.1 | ± | 0.1 |
| MCHC (g/dl) | 31.2 | ± | 0.8 | 30.7 | ± | 0.3 | 31.7 | ± | 0.7 | 31.1 | ± | 0.7 |
| CH (pg) | 12.1 | ± | 0.3 | 11.1 | ± | 0.4 | 11.8 | ± | 0.2 | 11.0 | ± | 0.6 |
| Reticulocytes (10^3^/μl) | 303 | ± | 11 | 282 | ± | 23 | 328 | ± | 15 | 312 | ± | 26 |
| Red cell count (10^12^/l) | 9.02 | ± | 0.12 | 8.63 | ± | 0.14 | 8.83 | ± | 0.11 | 8.52 | ± | 0.25 |
|  | Sham | | | TAC | | | Sham | | | TAC | | |
|  | (*n* = 8) | | | (*n* = 8) | | | (*n* = 8) | | | (*n* = 8) | | |

| Serum ferritin (ng/dl) | 86 | ± | 6 | 50 | ± | 5^*^ | 106 | ± | 15 | 63 | ± | 7^**^ |
| --- | --- | --- | --- | --- | --- | --- | --- | --- | --- | --- | --- | --- |
| Serum iron (mg/dl) | 138 | ± | 5 | 129 | ± | 9 | 151 | ± | 11 | 162 | ± | 18 |
| Transferrin saturation (%) | 50.3 | ± | 1.8 | 40.8 | ± | 1.9 | 45.7 | ± | 2.4 | 44.3 | ± | 3.9 |

PCV, packed cell volume; MCV, mean corpuscular volume; MCH, mean corpuscular hemoglobin; MCHC, mean corpuscular hemoglobin concentration; CH, hemoglobin concentration. Data are the mean ± SEM. *n* indicates the number of biologically independent samples. The data were evaluated by one-way analysis of variance (ANOVA), followed by Tukey–Kramer’s post hoc test. ^*^*P* = 0.0418 and ^**^*P* = 0.0108 versus the corresponding sham-operated group. Exact *P* values are provided in Supplementary file 1. Source Data file is provided in Figure 4—source data 3.
