# Supplementary material for: Iron derived from autophagy-mediated ferritin degradation induces cardiomyocyte death and heart failure in mice
Source: eLife. 2021 Feb 2;10:e62174. doi: 10.7554/eLife.62174 (PMC7853718; doi:10.7554/eLife.62174)
Supplement: Supplementary file 1. — The number of independent biological repeats (n) is shown in the figure legends. P values are shown below. [file elife-62174-supp1.docx]

**Supplementary file 1. Quantification and statistical analysis.**

The number of independent biological repeats (*n*) is shown in the figure legends.

*P* values are shown below.

|  |  | Sham-*Ncoa4*^+/+^ | Sham-*Ncoa4*^–/–^ | Sham-*Ncoa*4^+/+^ | TAC-*Ncoa4*^+/+^ |
| --- | --- | --- | --- | --- | --- |
|  |  | vs | vs | vs | vs |
|  |  | TAC*-Ncoa4^+/+^* | TAC*-*  *Ncoa4*^–/–^ | Sham-*Ncoa4*^–/–^ | TAC-  *Ncoa*4^–/–^ |
| Fig.1B | LVIDd | <0.0001 | >0.9999 | 0.7725 | <0.0001 |
|  | LVIDs | <0.0001 | 0.7698 | 0.9692 | <0.0001 |
|  | FS | <0.0001 | 0.2366 | 0.9925 | <0.0001 |
|  | IVSd | <0.0001 | <0.0001 | 0.9943 | 0.8523 |
|  | LVPWd | <0.0001 | <0.0001 | 0.9998 | 0.0781 |
|  | LV mass | <0.0001 | 0.0005 | 0.9671 | <0.0001 |
| Fig.1C | LV/Tibia | <0.0001 | 0.0353 | 0.9819 | 0.0002 |
|  | Lung/Tibia | 0.0357 | 0.9988 | 0.9856 | 0.0104 |
| Fig.1D | Fibrotic fraction | 0.0061 | 0.8162 | >0.9999 | 0.0341 |
|  | Cardiomyocyte area | <0.0001 | 0.0372 | 0.7510 | 0.0009 |
| Fig. 2A | FTH1/GAPDH | 0.0073 | 0.0025 | <0.0001 | <0.0001 |
| Fig. 2B | *Fth1/Gapdh* | 0.0211 | 0.1080 | 0.7994 | 0.3657 |
| Fig. 2C | See below |  |  |  |  |
| Fig. 2D | See below |  |  |  |  |
| Fig. 2F | See below |  |  |  |  |
| Fig. 2H | See below |  |  |  |  |
| Fig. 3A | FTH1/GAPDH | 0.0238 | >0.9999 | 0.1363 | 0.0002 |
| Fig. 3B | *Fth1/Gapdh* | <0.0001 | 0.9614 | 0.9765 | <0.0001 |
| Fig. 3D | LC3B-FTH1 | <0.0001 | 0.9526 | 0.6509 | <0.0001 |
| Fig. 3F | LAMP2a-FTH1 | 0.0008 | >0.9999 | 0.9130 | 0.0002 |
| Fig. 4A | Total non-heme iron | 0.0076 | <0.0001 | 0.9749 | 0.1052 |
|  | Fe^2+^ | 0.0424 | <0.0001 | 0.9698 | 0.0056 |
|  | Fe^3+^ | 0.0181 | 0.9797 | 0.9999 | 0.0472 |
|  | Fe^2+^/FTH1 | <0.0001 | 0.8963 | 0.0709 | <0.0001 |
| Fig. 4B | MDA | 0.0076 | 0.9935 | 0.9778 | 0.0101 |
| Fig. 4C | *Ptgs2/Gapdh* | <0.0001 | 0.0931 | 0.9867 | 0.0047 |
| Fig. 4D | Serum TnT | <0.0001 | 0.0019 | 0.9945 | 0.0002 |
| Fig. 4E | GPX4/GAPDH | 0.0008 | 0.9688 | 0.9483 | 0.0077 |
| Fig. 4F | Total glutathione | 0.0277 | 0.7410 | 0.2926 | 0.9896 |
|  | GSH | 0.0193 | 0.7688 | 0.4957 | 0.7841 |
|  | GSSG | <0.0001 | 0.8898 | 0.9540 | 0.0005 |
|  | GSH/GSSG | <0.0001 | 0.4797 | 0.4464 | 0.0083 |
| Fig. 4G | Glutamine | 0.0019 | 0.8047 | 0.7773 | 0.0017 |
| Fig. 5 | See below |  |  |  |  |
| Fig. 6B | LVIDd | <0.0001 | 0.0110 | 0.9971 | 0.0001 |
|  | LVIDs | <0.0001 | 0.0137 | 0.9988 | <0.0001 |
|  | FS | <0.0001 | 0.0007 | 0.9975 | <0.0001 |
|  | IVSd | <0.0001 | <0.0001 | 0.9999 | <0.0001 |
|  | LVPWd | <0.0001 | <0.0001 | 0.9974 | <0.0001 |
|  | LV mass | <0.0001 | 0.0036 | 0.9995 | <0.0001 |
| Fig. 6C | LV/Tibia | <0.0001 | 0.0058 | 0.9997 | 0.0013 |
|  | Lung/Tibia | 0.2944 | 0.9265 | >0.9999 | 0.6077 |
| Fig. 6D | Fibrotic fraction | 0.0040 | 0.8957 | 0.9597 | 0.0061 |
|  | Cardiomyocyte area | <0.0001 | 0.0073 | 0.9043 | 0.0002 |
| Fig. 6E | MDA | <0.0001 | 0.4018 | 0.4598 | <0.0001 |
| Fig. 6F | *Ptgs2/Gapdh* | <0.0001 | 0.0553 | 0.9866 | 0.0007 |
| Fig. 1 sup 3 | *Nppa/Gapdh* | 0.0003 | 0.8650 | >0.9999 | 0.0024 |
|  | *Nppb/Gapdh* | <0.0001 | 0.5173 | 0.7589 | 0.0385 |
|  | *Myh7/Gapdh* | 0.0011 | 0.6632 | 0.9998 | 0.0168 |
|  | *Col1a2/Gapdh* | 0.0016 | 0.7612 | 0.9992 | 0.0262 |
|  | *Col3a1/Gapdh* | 0.0014 | 0.7403 | 0.9783 | 0.0475 |
| Fig. 1 sup 4 | See below |  |  |  |  |
| Fig. 2 sup 1 | FS pre | >0.9999 | 0.9999 | 0.9930 | 0.9914 |
|  | FS Day3 | 0.0014 | 0.0002 | 0.8648 | 0.5522 |
|  | FS Day5 | <0.0001 | 0.9986 | 0.7090 | <0.0001 |
|  | FS Day7 | 0.0002 | 0.8770 | >0.9999 | 0.0004 |
|  | LVIDd pre | 0.7152 | 0.5719 | 0.3223 | 0.2317 |
|  | LVIDd Day3 | 0.0203 | 0.5512 | 0.8783 | 0.3627 |
|  | LVIDd Day5 | 0.0113 | 0.1788 | 0.3835 | 0.0250 |
|  | LVIDd Day7 | 0.0003 | 0.7587 | 0.4078 | 0.0052 |
|  | LVIDs pre | 0.8027 | 0.6817 | 0.5148 | 0.4013 |
|  | LVIDs Day3 | 0.0057 | 0.0407 | 0.9781 | 0.7071 |
|  | LVIDs Day5 | <0.0001 | 0.3945 | 0.9285 | <0.0001 |
|  | LVIDs Day7 | <0.0001 | 0.6581 | 0.8404 | <0.0001 |
| Fig. 4 source data 2 | Hemoglobin | 0.4000 | 0.5128 | 0.9988 | >0.9999 |
|  | PCV | 0.8363 | 0.9329 | 0.9452 | 0.9896 |
|  | MCV | 0.9741 | 0.9741 | 0.9998 | 0.9998 |
|  | MCH | 0.9795 | 0.9997 | 0.4255 | 0.5957 |
|  | MCHC | 0.9522 | 0.9374 | 0.9567 | 0.9685 |
|  | CH | 0.3350 | 0.5384 | 0.9449 | 0.9978 |
|  | Reticulocytes | 0.8666 | 0.9330 | 0.8102 | 0.7126 |
|  | Red cell count | 0.3543 | 0.5567 | 0.8305 | 0.9613 |
|  | Serum ferritin | 0.0418 | 0.0108 | 0.4140 | 0.7553 |
|  | Serum iron | 0.9487 | 0.9060 | 0.8667 | 0.2178 |
|  | Transferrin saturation | 0.0724 | 0.9790 | 0.6122 | 0.7875 |
| Fig. 4 sup 2A | Total non-heme iron | 0.0003 | 0.0378 | 0.9991 | 0.1301 |
| Fig. 4 sup 2B | IL-6 | <0.0001 | <0.0001 | 0.7517 | <0.0001 |
| Fig. 4 sup 3A | IREB2/GAPDH | 0.4012 | 0.4110 | 0.2590 | 0.2517 |
|  | SLC40A1/GAPDH | 0.1798 | 0.9553 | >0.9999 | 0.3750 |
|  | TFRC/GAPDH | 0.3555 | 0.1378 | 0.9917 | 0.8194 |
| Fig. 4 sup 3B | *Tfrc/Gapdh* | >0.9999 | 0.9995 | 0.8453 | 0.9102 |
|  | *Slc11a2/Gapdh* | 0.1320 | 0.9951 | 0.0086 | 0.4759 |
|  | *Cdc14a/Gapdh* | 0.8031 | 0.0134 | 0.9984 | 0.1399 |
|  | *Cdc42bpa/Gapdh* | 0.2606 | 0.9999 | 0.2856 | >0.9999 |
|  | *Cxcl16/Gapdh* | 0.0224 | 0.9999 | 0.7401 | 0.0014 |
| Fig. 4 sup 3C | IRE binding activity | 0.6966 | 0.9649 | 0.8343 | 0.8828 |
| Fig. 4 sup 4A | 4-HNE | 0.0044 | 0.9845 | 0.9973 | 0.0061 |
| Fig. 4 sup 4B | SOD2/GAPDH | 0.4080 | 0.6391 | 0.5983 | 0.4450 |
|  | HO-1/GAPDH | 0.4301 | 0.9994 | >0.9999 | 0.5209 |
| Fig. 4 sup 5A | *Slc7a11/Gapdh* | 0.2420 | 0.5814 | 0.8656 | 0.5016 |
| Fig. 4 sup 5B | Cystine | 0.8404 | 0.2799 | 0.0197 | 0.8443 |
| Fig. 4 sup 5C | Glutamate | <0.0001 | 0.2402 | 0.5598 | <0.0001 |
| Fig. 4 sup 5D | *Slc38a1/Gapdh* | 0.4451 | 0.4812 | <0.0001 | 0.0743 |
|  | *Slc1a5/Gapdh* | 0.9319 | 0.8393 | 0.2264 | 0.3284 |
|  | *Slc7a5/Gapdh* | 0.8747 | 0.0088 | 0.0032 | 0.6629 |
|  | *Gls1/Gapdh* | 0.0033 | 0.8790 | 0.0073 | 0.9733 |
|  | *Gls2/Gapdh* | 0.8001 | 0.8752 | 0.9883 | 0.9984 |
| Fig. 5 sup 1 | See below |  |  |  |  |
| Fig. 5 sup 2 | See below |  |  |  |  |
| Fig. 6 sup 1 | See below table |  |  |  |  |
| Fig. 6 sup 2A | See below |  |  |  |  |
| Fig. 6 sup 2B | See below |  |  |  |  |

Fig. 2C

*P* = 0.2329 (Day 3), 0.0018 (Day 5), 0.0002 (Day7), and 0.0001 (4 weeks) versus sham.

Fig. 2D

*P* = 0.9983 (Day 3), <0.0001 (Day 5), 0.0002 (Day7), and 0.0051 (4 weeks) versus sham.

Fig. 2F

*P* = 0.9894 (Day 3), <0.0001 (Day 5), <0.0001 (Day7), and <0.0001 (4 weeks) versus sham.

Fig. 2H

*P* = 0.8986 (Day 3), 0.1286 (Day 5), 0.0003 (Day 7), and 0.4934 (4 weeks) versus sham.

Fig. 5A

*P* = >0.9999 (*Ncoa4*^+/+^;Iso (0);Fer(+)), <0.0001 (*Ncoa4*^+/+^;Iso (10);Fer(-)), 0.8748 (*Ncoa4*^+/+^;Iso (10);Fer(+)), <0.0001 (*Ncoa4*^+/+^;Iso (100);Fer(-)), 0.0098 (*Ncoa4*^+/+^;Iso (100);Fer(+)), 0.9998 (*Ncoa4*^–/–^;Iso (0);Fer(-)), >0.9999 (*Ncoa4*^–/–^;Iso (0);Fer(+)), 0.9790 (*Ncoa4*^–/–^;Iso (10);Fer(-)), 0.9882 (*Ncoa4*^–/–^;Iso (10);Fer(+)), 0.5008 (*Ncoa4*^–/–^;Iso (100);Fer(-)), 0.3011 (*Ncoa4*^–/–^ ;Iso (100);Fer(+)) versus *Ncoa4*^+/+^;Iso (0);Fer(-).

*P* = >0.9999 (*Ncoa4*^+/+^;Iso (0);Fer(+)), 0.0154 (*Ncoa4*^+/+^;Iso (10);Fer(+)), 0.0003 (*Ncoa4*^+/+^;Iso (100);Fer(+)), >0.9999 (*Ncoa4*^–/–^;Iso (0);Fer(+)), >0.9999 (*Ncoa4*^–/–^;Iso (10);Fer(+)), >0.9999 (*Ncoa4*^–/–^;Iso (100);Fer(+)) versus the corresponding ferrostatin-1 non treated group.

*P* = 0.9998 (*Ncoa4*^–/–^;Iso (0);Fer(-)), 0.9993 (*Ncoa4*^–/–^;Iso (0);Fer(+)), 0.0048 (*Ncoa4*^–/–^;Iso (10);Fer(-)), >0.9999 (*Ncoa4*^–/–^;Iso (10);Fer(+)), <0.0001 (*Ncoa4*^–/–^;Iso (100);Fer(-)), 0.9553 (*Ncoa4*^–/–^;Iso (100);Fer(+)) versus the corresponding *Ncoa4*^+/+^ group.

Fig. 5B

*P* = >0.9999 (*Ncoa4*^+/+^;Iso (0);Fer(+)), 0.0002 (*Ncoa4*^+/+^;Iso (10);Fer(-)), 0.9963 (*Ncoa4*^+/+^;Iso (10);Fer(+)), <0.0001 (*Ncoa4*^+/+^;Iso (100);Fer(-)), 0.9359 (*Ncoa4*^+/+^;Iso (100);Fer(+)), >0.9999 (*Ncoa4*^–/–^;Iso (0);Fer(-)), >0.9999 (*Ncoa4*^–/–^;Iso (0);Fer(+)), 0.9989 (*Ncoa4*^–/–^;Iso (10);Fer(-)), >0.9999 (*Ncoa4*^–/–^;Iso (10);Fer(+)), 0.9941 (*Ncoa4*^–/–^;Iso (100);Fer(-)), >0.9999 (*Ncoa4*^–/–^;Iso (100);Fer(+)) versus *Ncoa4*^+/+^;Iso (0);Fer(-).

*P* = >0.9999 (*Ncoa4*^+/+^;Iso (0);Fer(+)), 0.0054 (*Ncoa4*^+/+^;Iso (10);Fer(+)), <0.0001 (*Ncoa4*^+/+^;Iso (100);Fer(+)), >0.9999 (*Ncoa4*^–/–^;Iso (0);Fer(+)), >0.9999 (*Ncoa4*^–/–^;Iso (10);Fer(+)), 0.9965 (*Ncoa4*^–/–^;Iso (100);Fer(+)) versus the corresponding ferrostatin-1 non treated group.

*P* = >0.9999 (*Ncoa4*^–/–^;Iso (0);Fer(-)), >0.9999 (*Ncoa4*^–/–^;Iso (0);Fer(+)), 0.0036 (*Ncoa4*^–/–^;Iso (10);Fer(-)), 0.9996 (*Ncoa4*^–/–^;Iso (10);Fer(+)), <0.0001 (*Ncoa4*^–/–^;Iso (100);Fer(-)), 0.9528 (*Ncoa4*^–/–^;Iso (100);Fer(+)) versus the corresponding *Ncoa4*^+/+^ group.

Fig. 5C

*P* = >0.9999 (*Ncoa4*^+/+^;Iso (0);Fer(+)), 0.0202 (*Ncoa4*^+/+^;Iso (10);Fer(-)), 0.8828 (*Ncoa4*^+/+^;Iso (10);Fer(+)), <0.0001 (*Ncoa4*^+/+^;Iso (100);Fer(-)), 0.2178 (*Ncoa4*^+/+^;Iso (100);Fer(+)), 0.9998 (*Ncoa4*^–/–^;Iso (0);Fer(-)), >0.9999 (*Ncoa4*^–/–^;Iso (0);Fer(+)), 0.6057 (*Ncoa4*^–/–^;Iso (10);Fer(-)), >0.9999 (*Ncoa4*^–/–^;Iso (10);Fer(+)), 0.1747 (*Ncoa4*^–/–^;Iso (100);Fer(-)), 0.9985 (*Ncoa4*^–/–^;Iso (100);Fer(+)) versus *Ncoa4*^+/+^;Iso (0);Fer(-).

*P* = >0.9999 (*Ncoa4*^+/+^;Iso (0);Fer(+)), 0.6074 (*Ncoa4*^+/+^;Iso (10);Fer(+)), <0.0001 (*Ncoa4*^+/+^;Iso (100);Fer(+)), >0.9999 (*Ncoa4*^–/–^;Iso (0);Fer(+)), 0.6470 (*Ncoa4*^–/–^;Iso (10);Fer(+)), 0.7076 (*Ncoa4*^–/–^;Iso (100);Fer(+)) versus the corresponding ferrostatin-1 non-treated group.

*P* = 0.9998 (*Ncoa4*^–/–^;Iso (0);Fer(-)), >0.9999 (*Ncoa4*^–/–^;Iso (0);Fer(+)), 0.8839 (*Ncoa4*^–/–^;Iso (10);Fer(-)), 0.9064 (*Ncoa4*^–/–^;Iso (10);Fer(+)), 0.0001 (*Ncoa4*^–/–^;Iso (100);Fer(-)), 0.7740 (*Ncoa4*^–/–^;Iso (100);Fer(+)) versus the corresponding *Ncoa4*^+/+^ group.

Fig. 5D

*P* = >0.9999 (*Ncoa4*^+/+^;Iso (0);Fer(+)), 0.0890 (*Ncoa4*^+/+^;Iso (10);Fer(-)), 0.9978 (*Ncoa4*^+/+^;Iso (10);Fer(+)), <0.0001 (*Ncoa4*^+/+^;Iso (100);Fer(-)), 0.3011 (*Ncoa4*^+/+^;Iso (100);Fer(+)), >0.9999 (*Ncoa4*^–/–^;Iso (0);Fer(-)), >0.9999 (*Ncoa4*^–/–^;Iso (0);Fer(+)), >0.9999 (*Ncoa4*^–/–^;Iso (10);Fer(-)), >0.9999 (*Ncoa4*^–/–^;Iso (10);Fer(+)), 0.9539 (*Ncoa4*^–/–^;Iso (100);Fer(-)), >0.9999 (*Ncoa4*^–/–^;Iso (100);Fer(+)) versus *Ncoa4*^+/+^;Iso (0);Fer(-).

*P* = >0.9999 (*Ncoa4*^+/+^;Iso (0);Fer(+)), 0.5341 (*Ncoa4*^+/+^;Iso (10);Fer(+)), <0.0001 (*Ncoa4*^+/+^;Iso (100);Fer(+)), >0.9999 (*Ncoa4*^–/–^;Iso (0);Fer(+)), >0.9999 (*Ncoa4*^–/–^;Iso (10);Fer(+)), 0.9915 (*Ncoa4*^–/–^;Iso (100);Fer(+)) versus the corresponding ferrostatin-1 non-treated group.

*P* = >0.9999 (*Ncoa4*^–/–^;Iso (0);Fer(-)), >0.9999 (*Ncoa4*^–/–^;Iso (0);Fer(+)), 0.3406 (*Ncoa4*^–/–^;Iso (10);Fer(-)), 0.9993 (*Ncoa4*^–/–^;Iso (10);Fer(+)), <0.0001 (*Ncoa4*^–/–^;Iso (100);Fer(-)), 0.4730 (*Ncoa4*^–/–^;Iso (100);Fer(+)) versus the corresponding *Ncoa4*^+/+^ group.

Fig. 5E

*P* = 0.0212 (*Ncoa4*^+/+^;Iso (10)), 0.0002 (*Ncoa4*^+/+^;Iso (100)), 0.0086 (*Ncoa4*^–/–^;Iso (0)), 0.0040 (*Ncoa4*^–/–^;Iso (10)), 0.0481 (*Ncoa4*^–/–^;Iso (100)) versus *Ncoa4*^+/+^;Iso (0).

*P* = 0.0086 (*Ncoa4*^–/–^;Iso (0)), <0.0001 (*Ncoa4*^–/–^;Iso (10)), <0.0001 (*Ncoa4*^–/–^;Iso (100)) versus the corresponding *Ncoa4*^+/+^ group.

Fig. 1 sup 4

*P* = 0.3154 (LVIDd), 0.5175 (LVIDs), 0.7037 (FS)

Fig. 5 sup 1A

*P* = >0.9999 (*Ncoa4*^+/+^;Era (0);Fer(+)), 0.0031 (*Ncoa4*^+/+^;Era (10);Fer(-)), 0.7365 (*Ncoa4*^+/+^;Era (10);Fer(+)), <0.0001 (*Ncoa4*^+/+^;Era (20);Fer(-)), 0.0319 (*Ncoa4*^+/+^;Era (20);Fer(+)), 0.8246 (*Ncoa4*^–/–^;Era (0);Fer(-)), 0.9926 (*Ncoa4*^–/–^;Era (0);Fer(+)), 0.9969 (*Ncoa4*^–/–^;Era (10);Fer(-)), >0.9999 (*Ncoa4*^–/–^;Era (10);Fer(+)), 0.0404 (*Ncoa4*^–/–^;Era (20);Fer(-)), 0.9607 (*Ncoa4*^–/–^;Era (20);Fer(+)) versus *Ncoa4*^+/+^;Era (0);Fer(-).

*P* = >0.9999 (*Ncoa4*^+/+^;Era (0);Fer(+)), 0.3789 (*Ncoa4*^+/+^;Era (10);Fer(+)), <0.0001 (*Ncoa4*^+/+^;Era (20);Fer(+)), >0.9999 (*Ncoa4*^–/–^;Era (0);Fer(+)), 0.9990 (*Ncoa4*^–/–^ ;Era (10);Fer(+)), 0.6052 (*Ncoa4*^–/–^;Era (20);Fer(+)) versus the corresponding ferrostatin-1 non treated group.

*P* = 0.8246 (*Ncoa4*^–/–^;Era (0);Fer(-)), 0.9412 (*Ncoa4*^–/–^;Era (0);Fer(+)), 0.0544 (*Ncoa4*^–/–^;Era (10);Fer(-)), 0.8051 (*Ncoa4*^–/–^;Era (10);Fer(+)), <0.0001 (*Ncoa4*^–/–^;Era (20);Fer(-)), 0.5450 (*Ncoa4*^–/–^;Era (20);Fer(+)) versus the corresponding *Ncoa4*^+/+^ group.

Fig. 5 sup 1B

*P* = >0.9999 (*Ncoa4*^+/+^;Era (0);Fer(+)), 0.0005 (*Ncoa4*^+/+^;Era (10);Fer(-)), >0.9999 (*Ncoa4*^+/+^;Era (10);Fer(+)), <0.0001 (*Ncoa4*^+/+^;Era (20);Fer(-)), 0.9934 (*Ncoa4*^+/+^;Era (20);Fer(+)), >0.9999 (*Ncoa4*^–/–^;Era (0);Fer(-)), >0.9999 (*Ncoa4*^–/–^;Era (0);Fer(+)), 0.9991 (*Ncoa4*^–/–^;Era (10);Fer(-)), 0.9992 (*Ncoa4*^–/–^;Era (10);Fer(+)), 0.9878 (*Ncoa4*^–/–^;Era (20);Fer(-)), >0.9999 (*Ncoa4*^–/–^;Era (20);Fer(+)) versus *Ncoa4*^+/+^;Era (0).

*P* = >0.9999 (*Ncoa4*^+/+^;Era (0);Fer(+)), 0.0003 (*Ncoa4*^+/+^;Era (10);Fer(+)), <0.0001 (*Ncoa4*^+/+^;Era (20);Fer(+)), >0.9999 (*Ncoa4*^–/–^;Era (0);Fer(+)), >0.9999 (*Ncoa4*^–/–^;Era (10);Fer(+)), 0.9997 (*Ncoa4*^–/–^;Era (20);Fer(+)) versus the corresponding ferrostatin-1 non treated group.

*P* = >0.9999 (*Ncoa4*^–/–^;Era (0);Fer(-)), >0.9999 (*Ncoa4*^–/–^ ;Era (0);Fer(+)), 0.0086 (*Ncoa4*^–/–^;Era (10);Fer(-)), 0.9949 (*Ncoa4*^–/–^;Era (10);Fer(+)), <0.0001 (*Ncoa4*^–/–^;Era (20);Fer(-)), >0.9999 (*Ncoa4*^–/–^;Era (20);Fer(+)) versus the corresponding *Ncoa4*^+/+^ group.

Fig. 5 sup 1C

*P* = >0.9999 (*Ncoa4*^+/+^;Era (0);Fer(+)), 0.0516 (*Ncoa4*^+/+^;Era (10);Fer(-)), 0.9832 (*Ncoa4*^+/+^;Era (10);Fer(+)), <0.0001 (*Ncoa4*^+/+^;Era (20);Fer(-)), 0.9559 (*Ncoa4*^+/+^;Era (20);Fer(+)), >0.9999 (*Ncoa4*^–/–^;Era (0);Fer(-)), 0.9996 (*Ncoa4*^–/–^;Era (0);Fer(+)), >0.9999 (*Ncoa4*^–/–^;Era (10);Fer(-)), >0.9999 (*Ncoa4*^–/–^;Era (10);Fer(+)), 0.9845 (*Ncoa4*^–/–^;Era (20);Fer(-)), >0.9999 (*Ncoa4*^–/–^;Era (20);Fer(+)) versus *Ncoa4*^+/+^;Era (0);Fer(-).

*P* = >0.9999 (*Ncoa4*^+/+^;Era (0);Fer(+)), 0.5665 (*Ncoa4*^+/+^;Era (10);Fer(+)), <0.0001 (*Ncoa4*^+/+^;Era (20);Fer(+)), 0.9995 (*Ncoa4*^–/–^;Era (0);Fer(+)), 0.9899 (*Ncoa4*^–/–^;Era (10);Fer(+)), 0.8641 (*Ncoa4*^–/–^;Era (20);Fer(+)) versus the corresponding ferrostatin-1 non-treated group.

*P* = >0.9999 (*Ncoa4*^–/–^;Era (0);Fer(-)), 0.9998 (*Ncoa4*^–/–^;Era (0);Fer(+)), 0.2324 (*Ncoa4*^–/–^;Era (10);Fer(-)), 0.8483 (*Ncoa4*^–/–^;Era (10);Fer(+)), <0.0001 (*Ncoa4*^–/–^;Era (20);Fer(-)), 0.7660 (*Ncoa4*^–/–^;Era (20);Fer(+)) versus the corresponding *Ncoa4*^+/+^ group.

Fig. 5 sup 1D

*P* = >0.9999 (*Ncoa4*^+/+^;Era (0);Fer(+)), 0.3551 (*Ncoa4*^+/+^;Era (10);Fer(-)), >0.9999 (*Ncoa4*^+/+^;Era (10);Fer(+)), <0.0001 (*Ncoa4*^+/+^;Era (20);Fer(-)), 0.7060 (*Ncoa4*^+/+^;Era (20);Fer(+)), 0.9988 (*Ncoa4*^–/–^;Era (0);Fer(-)), 0.9994 (*Ncoa4*^–/–^;Era (0);Fer(+)), >0.9999 (*Ncoa4*^–/–^;Era (10);Fer(-)), 0.9996 (*Ncoa4*^–/–^;Era (10);Fer(+)), >0.9999 (*Ncoa4*^–/–^;Era (20);Fer(-)), >0.9999 (*Ncoa4*^–/–^;Era (20);Fer(+)) versus *Ncoa4*^+/+^;Era (0);Fer(-).

*P* = >0.9999 (*Ncoa4*^+/+^;Era (0);Fer(+)), 0.7829 (*Ncoa4*^+/+^;Era (10);Fer(+)), 0.0196 (*Ncoa4*^+/+^;Era (20);Fer(+)), >0.9999 (*Ncoa4*^–/–^;Era (0);Fer(+)), >0.9999 (*Ncoa4*^–/–^;Era (10);Fer(+)), 0.9998 (*Ncoa4*^–/–^;Era (20);Fer(+)) versus the corresponding ferrostatin-1 non-treated group.

*P* = 0.9988 (*Ncoa4*^–/–^;Era (0);Fer(-)), 0.9962 (*Ncoa4*^–/–^;Era (0);Fer(+)), 0.1861 (*Ncoa4*^–/–^;Era (10);Fer(-)), 0.9416 (*Ncoa4*^–/–^;Era (10);Fer(+)), 0.0002 (*Ncoa4*^–/–^;Era (20);Fer(-)), 0.4901 (*Ncoa4*^–/–^;Era (20);Fer(+)) versus the corresponding *Ncoa4*^+/+^ group.

Fig. 5 sup 2

*P* = 0.9997 (*Ncoa4*^+/+^;RSL3 (0);Fer(+)), 0.0002 (*Ncoa4*^+/+^;RSL3 (2);Fer(-)), 0.9904 (*Ncoa4*^+/+^; RSL3 (2);Fer(+)), <0.0001 (*Ncoa4*^+/+^; RSL3 (5);Fer(-)), 0.3195 (*Ncoa4*^+/+^; RSL3 (5);Fer(+)), 0.9762 (*Ncoa4*^–/–^; RSL3 (0);Fer(-)), 0.9999 (*Ncoa4*^–/–^; RSL3 (0);Fer(+)), 0.9997 (*Ncoa4*^–/–^; RSL3 (2);Fer(-)), >0.9999 (*Ncoa4*^–/–^; RSL3 (2);Fer(+)), >0.9999 (*Ncoa4*^–/–^; RSL3 (5);Fer(-)), 0.9981 (*Ncoa4*^–/–^; RSL3 (5);Fer(+)) versus *Ncoa4*^+/+^; RSL3 (0);Fer(-).

*P* = 0.9997 (*Ncoa4*^+/+^; RSL3 (0);Fer(+)), 0.0074 (*Ncoa4*^+/+^; RSL3 (2);Fer(+)), <0.0001 (*Ncoa4*^+/+^; RSL3 (5);Fer(+)), >0.9999 (*Ncoa4*^–/–^; RSL3 (0);Fer(+)), >0.9999 (*Ncoa4*^–/–^ ; RSL3 (2);Fer(+)), >0.9999 (*Ncoa4*^–/–^; RSL3 (5);Fer(+)) versus the corresponding ferrostatin-1 non treated group.

*P* = 0.9762 (*Ncoa4*^–/–^; RSL3 (0);Fer(-)), 0.9347 (*Ncoa4*^–/–^; RSL3 (0);Fer(+)), <0.0001 (*Ncoa4*^–/–^; RSL3 (2);Fer(-)), 0.9466 (*Ncoa4*^–/–^; RSL3 (2);Fer(+)), <0.0001 (*Ncoa4*^–/–^; RSL3 (5);Fer(-)), 0.0391 (*Ncoa4*^–/–^; RSL3 (5);Fer(+)) versus the corresponding *Ncoa4*^+/+^ group.

Fig. 6 sup 2A

*P* = 0.7698 (LVIDd), 0.7610 (LVIDs), 0.8111 (FS), 0.8527 (IVSd), 0.8759 (LVWPd), 0.8124 (LV mass)

Fig. 6 sup 2B

*P* = 0.2611 (LV/Tibia), 0.5477 (Lung/Tibia)

|  |  | Sham-saline | Sham-Fer-1 | Sham-Saline | TAC-Saline |
| --- | --- | --- | --- | --- | --- |
|  |  | vs | vs | vs | vs |
|  |  | TAC-saline | TAC-Fer-1 | Sham-Fer-1 | TAC-Fer-1 |
| Fig. 6 sup 1A | Nppa/Gapdh | 0.0009 | 0.8879 | >0.9999 | 0.0055 |
|  | *Nppb/Gapdh* | 0.0003 | 0.1828 | 0.9981 | 0.0396 |
|  | Myh7/Gapdh | <0.0001 | 0.2896 | 0.9982 | 0.0032 |
|  | Col1a21/Gapdh | <0.0001 | 0.0219 | 0.6727 | 0.0005 |
|  | Col3a1/Gapdh | 0.0003 | 0.1528 | 0.9232 | 0.0190 |
| Fig. 6 sup 1B | 4-HNE | 0.0011 | 0.3644 | 0.9855 | 0.0164 |
